# Sebelipase Alfa Improves Aminotransferase Levels in Lysosomal Acid Lipase Deficiency: Data From an International Registry

**DOI:** 10.1111/liv.70279

**Published:** 2025-08-08

**Authors:** Lorenzo D'Antiga, Jennifer Evans, Emilio Ros, Florian Abel, Manisha Balwani, Don P. Wilson, William Balistreri

**Affiliations:** ^1^ Department of Medicine and Surgery University of Milano–Bicocca Milan Italy; ^2^ Pediatric Hepatology, Gastroenterology and Transplantation Hospital Papa Giovanni XXIII Bergamo Italy; ^3^ Department of Epidemiology, Alexion, AstraZeneca Rare Disease Boston Massachusetts USA; ^4^ Institut D'investigacions Biomèdiques August Pi Sunyer (IDIBAPS), hospital Clinic Barcelona Spain; ^5^ Department of Global Medical Affairs, Alexion, AstraZeneca Rare Disease Boston Massachusetts USA; ^6^ Department of Genetics and Genomic Sciences Icahn School of Medicine at Mount Sinai New York New York USA; ^7^ Endocrinology Cook Children's Medical Center Fort Worth Texas USA; ^8^ UC Department of Pediatrics Cincinnati Children's Hospital Medical Center Cincinnati Ohio USA

**Keywords:** alanine aminotransferase, aspartate aminotransferase, enzyme replacement therapy, lysosomal acid lipase deficiency

## Abstract

**Background and Aims:**

In patients with lysosomal acid lipase deficiency (LAL‐D), elevations in alanine and aspartate aminotransferases (ALT, AST) are associated with liver damage. The objective of this analysis was to evaluate aminotransferase levels in patients treated with sebelipase alfa enzyme replacement therapy and untreated patients.

**Methods:**

Patients in this observational study were from the International LAL‐D Registry and included untreated and treated patients who started sebelipase alfa at age ≥ 6 months. Patients in the longitudinal analysis must have had baseline and longitudinal measures of ALT or AST. A subanalysis was performed on data from patients with 3 consecutive annual follow‐up measures.

**Results:**

Of 186 patients included in this analysis, 125 were treated with sebelipase alfa. Median age at diagnosis was 9.1 y for treated patients and 20.6 y for untreated patients. Baseline ALT levels were above the upper limit of normal in 93% of children aged < 18 years and 68% of adults aged ≥ 18 years. Among 53 treated patients who had ALT results reported at 3 consecutive annual measurements, 22 (42%) had ALT levels within normal limits after 1 year of sebelipase alfa treatment. Results were similar for AST. A repeated measures regression model of data from treated patients showed significant reductions from baseline in both ALT and AST levels across 3 years of the study. Untreated patients had no appreciable changes in aminotransferase elevations over time.

**Conclusions:**

Aminotransferase levels were elevated in most patients with LAL‐D at baseline. There were sustained improvements with sebelipase alfa treatment.

**Trial Registration:**

International LAL‐D Registry: NCT01633489, EUPAS13276

**Abbreviations**
AE: adverse event
ALT: alanine aminotransferase
AST: aspartate aminotransferase
ERT: enzyme‐replacement therapy
LAL‐D: lysosomal acid lipase deficiency
Nobs: number of observations
Q1, Q3: first and third quarters
SAE: serious adverse event
ULN: upper limit of normal
WNL: within normal limits

**Summary**

- Liver damage is common among people with lysosomal acid lipase deficiency (LAL‐D), especially those who are diagnosed with the disease early in life.
- Enzyme replacement therapy with sebelipase alfa can normalise blood markers of liver damage, suggesting that the treatment helps to protect the liver in these patients.
- Since liver damage can be life‐threatening, protecting the liver with sebelipase alfa treatment may improve outcomes in people with LAL‐D.

## 1 Introduction

Lysosomal acid lipase deficiency (LAL‐D) is a rare autosomal recessive lysosomal storage disorder caused by pathogenic variants in *LIPA*, resulting in diminished or absent LAL activity [1, 2, 3]. Loss of LAL activity leads to lysosomal accumulation of the LAL substrates triglyceride and cholesteryl ester in organs and tissues, especially in the liver, spleen, gut, and cardiovascular system [1, 3, 4, 5]. The clinical manifestations of LAL‐D vary by age of symptom onset. Patients who manifest symptoms of LAL‐D in early infancy can present with rapidly progressive disease characterised by hepatosplenomegaly, abdominal distention, macrophage‐driven inflammation, vomiting, diarrhoea, and failure to thrive and typically do not survive beyond the first year of life [1, 3, 6]. Patients who manifest symptoms of LAL‐D in childhood and adulthood can present with hepatomegaly, elevated aminotransferase levels, liver dysfunction, lobular inflammation, elevated total and low‐density lipoprotein cholesterol levels, low high‐density lipoprotein cholesterol levels and, in some patients, accumulation of cholesterol ester and carotene in the intestinal mucosa [1, 3, 4, 7, 8, 9]. These clinical manifestations may progress over time to liver fibrosis, cirrhosis, and the need for liver transplantation, as well as premature atherosclerosis [1, 2, 10, 11]. The diagnosis of LAL‐D is often missed or delayed, which may be a result of a clinical picture that overlaps with that of other more common conditions, such as familial hypercholesterolaemia or metabolic dysfunction–associated steatotic liver disease [1, 2, 7, 8].

Sebelipase alfa (KANUMA, Alexion, AstraZeneca Rare Disease) is a human LAL enzyme‐replacement therapy (ERT) approved for treatment of LAL‐D in more than 30 countries, including the United States, Canada, Japan, Australia, Israel, Saudi Arabia, the United Kingdom, and the countries in the European Union [12, 13, 14, 15, 16, 17]. Infants with rapidly progressive LAL‐D treated with sebelipase alfa and adequate nutritional care survived and developed well compared with historical controls [18, 19, 20]. In children and adults with LAL‐D, sebelipase alfa treatment improved liver‐related disease measures such as the liver enzymes alanine aminotransferase (ALT) and aspartate aminotransferase (AST), liver volume, and liver fat content, as well as parameters of lipid metabolism, including low‐ and high‐density lipoprotein cholesterol levels [5, 21, 22].

The International LAL‐D Registry is an ongoing observational study initiated in 2013 to generate real‐world evidence of the natural history of LAL‐D [8]. Rare disease registries are powerful tools for assessing outcomes that are underreported in clinical trials owing to low patient numbers and providing valuable insights on the natural history of disease over time [23]. After the EU and US approval of sebelipase alfa in 2015, the registry was amended to include information on treatment status, effectiveness, and safety, further broadening the availability of LAL‐D data [8, 24]. Results from registry analyses showed that baseline ALT and AST elevations and symptom onset at an early age correspond with more severe liver damage, while the AST‐to‐platelet ratio index is a poor predictor of disease severity in patients with LAL‐D [8, 25]. The current analysis had two objectives: (1) describe ALT and AST among patients who were enrolled in the International LAL‐D Registry and were either untreated or treated with sebelipase alfa and (2) evaluate ALT and AST among treated and untreated patients at baseline and over 5 years of follow‐up.

## 2 Methods

### 2.1 Registry Eligibility

The International LAL‐D Registry is an ongoing, observational, multisite study to collect data on patients with LAL‐D, with the aim of improving patient outcomes [8]. The registry protocol was approved by the institutional review board, or equivalent, at each participating site and was conducted in accordance with all applicable government regulations, the Declaration of Helsinki, and the principles of the International Council for Harmonisation Guidelines for Good Clinical Practice. All patients enrolled in the registry, or their parent or guardian, provided informed consent and authorisation prior to submitting their data into the registry. The registry is sponsored by Alexion, AstraZeneca Rare Disease and is overseen by a scientific advisory board that includes medical experts with experience in LAL‐D research or patient care.

### 2.2 Study Design

The registry enrols patients of any age who have a confirmed diagnosis of LAL‐D, including living and deceased individuals regardless of whether they were treated with sebelipase alfa or untreated. The current analysis included patients who had a diagnosis of LAL‐D confirmed by *LIPA* genotype and/or low LAL enzyme activity; were alive at the time of registry enrollment; and had known sex, date of registry enrollment, sebelipase alfa treatment status (including date of treatment initiation if ever treated), and date of birth or age at registry enrollment. Patients in the longitudinal analysis were also required to have baseline and longitudinal measures of ALT and/or AST. Patients with rapidly progressive LAL‐D who initiated treatment with sebelipase alfa prior to 6 months of age and patients with prior participation in a clinical trial that included sebelipase alfa were excluded from this analysis so as to not introduce bias by including these patients' initial data in analyses from the registry. Adverse events were analysed among patients in the safety population, which included all patients enrolled in the registry who had ever been treated with sebelipase alfa. Patient data were analysed through a registry cutoff date of 2 October 2023.

### 2.3 Demographics and Disease Outcomes

Baseline demographic data were analysed for all patients included in the study. Baseline ALT and AST results were analysed by patient age and treatment status (treated or untreated with sebelipase alfa) and reported as multiples of the upper limit of normal (ULN) because ULNs are age‐ and sex‐specific and can vary by laboratory. Baseline for all analyses was defined as the date of first sebelipase alfa treatment for treated patients or the date of registry enrollment for those who were untreated. ALT and AST results were categorised as within normal limits (WNL; defined as ≤ ULN), >ULN to < 1.5 × ULN, ≥ 1.5 to < 2 × ULN, ≥ 2 to < 3 × ULN, or ≥ 3 × ULN. Patient age at baseline was categorised as 0 to < 6 years, 6 to < 12 years, 12 to < 18 years, and ≥ 18 years. An analysis was performed using data from patients in the “Fixed Complete Cohort” to measure changes in ALT and AST over time in patients with regular monitoring. The Fixed Complete Cohort included treated and untreated patients who had ALT or AST values available at baseline and at each of the 3 consecutive annual follow‐up measurements. An additional cohort analysis was performed using data from patients who had ALT and AST values recorded at baseline and at ≥ 1 time point over 5 years of follow‐up. All adverse events (AEs) and serious AEs (SAEs) were recorded for treated patients throughout registry follow‐up.

### 2.4 Statistical Analysis

Results are summarised as median and interquartile range for continuous variables and as count and percentage for discrete variables. A repeated measures analysis using a linear mixed model regression provided estimates of the change in ALT and AST multiples of normal at each timepoint compared to baseline. Covariates in this analysis included treatment timepoint, age at baseline, and genotype. This analysis was performed using data from treated patients who were above the ULN at baseline and who had complete measurements at baseline and at each of the 3 consecutive annual follow‐up visits. The number of patients with results available for analysis throughout follow‐up varies owing to the observational nature of the registry in which patient data are collected in a real‐world setting under routine clinical care.

## 3 Results

### 3.1 Study Population

Of the 291 patients with LAL‐D who did not have rapidly progressive disease and were enrolled in the registry as of 2 October 2023, a total of 186 were included in this analysis; 125 had ever been treated with sebelipase alfa (“treated” patients) and 61 had never been treated with sebelipase alfa (“untreated” patients) (Figure S1). The primary reason for exclusion from the current analysis was prior participation in a clinical trial that included sebelipase alfa (*n* = 58). Baseline characteristics of the study population are summarised in Table 1. About half of the patients in the analysis were male, and 93% were White. Median (quartile [Q]1, Q3) age at diagnosis was 10.3 (6.3, 21.1) years and was lower among treated patients (9.1 [5.9, 13.8]) than among those who were untreated (20.6 [8.5, 41.5]). Median (Q1, Q3) age at the time of registry enrollment was 14.4 (9.1, 26.4) years for all patients and was lower among treated patients (11.9 [7.6, 16.7]) than among those who were untreated (25.3 [18.9, 44.8]). Baseline ALT and AST levels also differed by treatment status. At baseline, ALT levels were above the ULN in 94% of treated patients and in 66% of untreated patients. Similarly, baseline AST levels were above the ULN in 87% of treated patients and in 57% of those who were untreated. Among patients in the overall study population whose *LIPA* genotype was analysed, 40% were homozygous for c.894G>A variants.

**TABLE 1:** Baseline patient characteristics by sebelipase alfa treatment status.

Description	Treated patients (*n* = 125)	Untreated patients (*n* = 61)	Study population (*N* = 186)
Male, *n* (%)	69 (55.2)	23 (37.7)	92 (49.5)
Patients with race data, *n* (%)	120 (96.0)	57 (93.4)	177 (95.2)
Race, *n* (%)			
White	111 (92.5)	53 (93.0)	164 (92.7)
Asian	3 (2.5)	1 (1.8)	4 (2.3)
Other/multiple	6 (5.0)	3 (5.3)	9 (5.1)
Unknown or missing race, n	5	4	9
Age at diagnosis, median (Q1, Q3), years	9.1 (5.9, 13.8)	20.6 (8.5, 41.5)	10.3 (6.3, 21.1)
Age at registry enrollment, median (Q1, Q3), years	11.9 (7.6, 16.7)	25.3 (18.9, 44.8)	14.4 (9.1, 26.4)
Patients with baseline ALT, *n* (%)	104 (83.2)	58 (95.1)	162 (87.1)
Baseline ALT>ULN^a^, *n* (%)	98 (94.2)	38 (65.5)	136 (84.0)
Patients with baseline AST, *n* (%)	102 (81.6)	49 (80.3)	151 (81.2)
Baseline AST>ULN^a^, *n* (%)	89 (87.3)	28 (57.1)	117 (77.5)
*LIPA* genotype data analysed, *n*	108	47	155
Homozygous for c.894G>A, *n* (%)	39 (36.1)	23 (48.9)	62 (40.0)
Homozygous for other variants, *n* (%)	20 (18.5)	6 (12.8)	26 (16.8)
Compound heterozygous for c.894G>A, *n* (%)	35 (32.4)	15 (31.9)	50 (32.3)
Compound heterozygous for other variants, *n* (%)	14 (13.0)	2 (4.3)	16 (10.3)

Abbreviations: ALT, alanine aminotransferase; AST, aspartate aminotransferase; Q, quartile; ULN, upper limit of normal.

^a^ ULN for ALT and AST vary by age and sex.

### 3.2 Baseline Aminotransferase Levels

**FIGURE 1:**
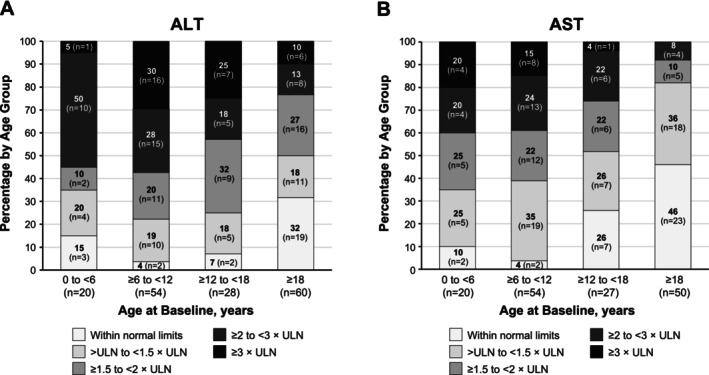
Baseline ALT (*n* = 162) and AST (*n* = 151) values within normal limits and in multiples of the ULN stratified by age. ALT, alanine aminotransferase; AST, aspartate aminotransferase; ULN, upper limit of normal.

Of the 186 patients in the analysis, 162 (87%) and 151 (81%) had available baseline ALT and AST data. Results were categorised as WNL or as multiples of the ULN and stratified by age group. ALT and AST levels were above the ULN for most patients in all age groups. However, the elevations were more common and more pronounced among paediatric patients than among adult patients. ALT levels were above the ULN in 93% of children aged < 18 years and in 68% of adults aged ≥ 18 years (Figure 1A). AST levels followed a similar pattern, in which levels were above the ULN in 89% and 54% of children and adults, respectively (Figure 1B).

When analysing age as a continuous variable, age at baseline was inversely associated with ALT (*r* = −0.281; *p* = 0.0003) and AST (*r* = −0.478; *p* < 0.0001) multiples of normal at baseline (Table 2). Genotype (c.[894G>A];[894G>A]) was significantly associated with ALT (*p* = 0.0194) and AST (*p* = 0.0096) multiples of normal at baseline (Table 2). There was no correlation between ALT or AST multiples of normal at baseline and sex (Table 2).

**TABLE 2:** Correlation analyses among all patients (treated or untreated) with baseline ALT or AST.

	*N*	Mean (SD)	*r*_*s*_	*p*
ALT multiple of normal				
Age at baseline	162	2.1 (1.26)	−0.281	0.0003
Sex				
Female	81	1.9 (1.20)	—	0.0837
Male	81	2.3 (1.30)	—	—
Genotype				
Homozygous for c.894G>A	57	2.0 (1.04)	—	0.0194
Homozygous for other variants	23	1.5 (0.86)	—	—
Compound heterozygous for c.894G>A	45	2.5 (1.70)	—	—
Compound heterozygous for other variants	14	2.5 (1.19)	—	—
AST multiple of normal				
Age at baseline	151	1.7 (0.94)	−0.478	< 0.0001
Sex				
Female	77	1.6 (0.97)	—	0.9001
Male	74	1.7 (0.91)	—	—
Genotype				
Homozygous for c.894G>A	56	1.5 (0.60)	—	0.0096
Homozygous for other variants	22	1.5 (1.13)	—	—
Compound heterozygous for c.894G>A	42	1.8 (0.98)	—	—
Compound heterozygous for other variants	14	2.3 (0.94)	—	—

Abbreviations: ALT, alanine aminotransferase; AST, aspartate aminotransferase.

### 3.3 Fixed Complete Cohort Analysis

A subanalysis was performed among patients in the Fixed Complete Cohort, which included patients who had measures of ALT or AST at baseline and at each of 3 consecutive annual follow‐ups. A total of 75 patients (53 treated and 22 untreated) had ALT results at all time points, and 70 patients (51 treated and 19 untreated) had AST results at all time points. The demographics of treated patients in this cohort who had baseline ALT or AST levels > ULN are summarised in Table S1. Demographics were similar to those of treated patients from the overall study population.

**FIGURE 2:**
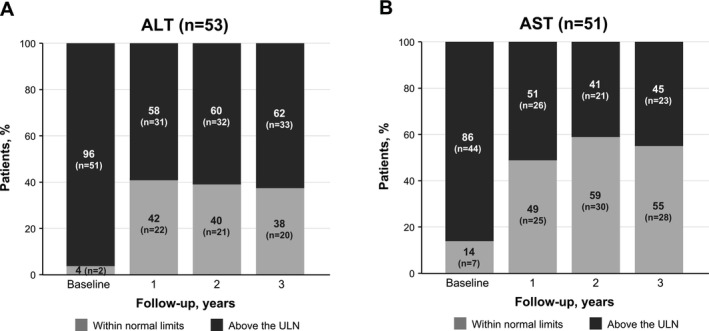
Proportion of treated patients with ALT and AST values WNL at baseline and at 3 consecutive annual follow‐up measures. ALT, alanine aminotransferase; AST, aspartate aminotransferase; ULN, upper limit of normal; WNL, within normal limits.

Of the 53 treated patients in the Fixed Complete Cohort with ALT data, at baseline, 51 (96%) had ALT levels above the ULN (Figure 2A). After 1 year of follow‐up, 22 of 53 (42%) treated patients had ALT levels WNL (Figure 2A). Improvements in ALT were sustained among these patients, with ALT levels WNL in 20 of 53 (38%) patients at 3 years of follow‐up. Median ALT levels significantly decreased by 51.4% (95% CI: −59.3, −46.2) after 1 year, with similar results found over 3 years of treatment. AST improvements in treated patients followed a similar trend. Of the 51 treated patients in the Fixed Complete Cohort with AST data, 44 (86%) had AST levels above the ULN at baseline (Figure 2B). AST levels were WNL in 25 of 51 (49%) and 28 of 51 (55%) treated patients at 1 year and 3 years of follow‐up, respectively. After 1 year of treatment, median AST levels decreased by 42.1% from baseline (95% CI: −49.0, −40.0), with similar results recorded throughout follow‐up. Among treated patients in the Fixed Complete Cohort, mean ALT and AST multiples of normal at baseline were not significantly different by age group at baseline (*p* = 0.3976 and 0.0729, respectively).

**FIGURE 3:**
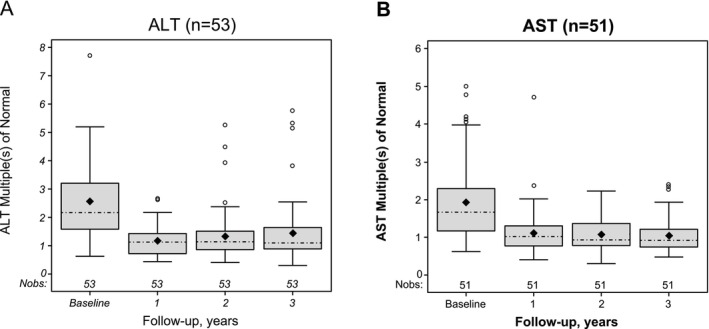
ALT and AST multiples of the ULN in patients treated with sebelipase alfa who had 3 consecutive annual follow‐up measures. Open circles represent outliers, black diamonds represent means, and dashed lines represent medians. ALT, alanine aminotransferase; AST, aspartate aminotransferase; Nobs, number of observations; ULN, upper limit of normal.

To account for the correlation of repeated measures within individuals, a linear mixed regression model was used to estimate the change in ALT and AST multiples of normal at each timepoint compared with baseline among treated patients in the Fixed Complete Cohort. ALT and AST decreased significantly from baseline at each follow‐up measure. In Year 1, ALT was lower by −1.4 multiples of normal (95% CI: −1.7, −1.0) and AST was lower by −0.8 multiples of normal (95% CI: −1.1, −0.5; Figure 3A,B). In Year 2, ALT was lower by −1.2 multiples of normal (95% CI: −1.5, −0.9) and AST was lower by −0.8 multiple of normal (95% CI: −1.1, −0.6); and in Year 3, ALT was lower by −1.1 multiples of normal (95% CI: −1.5, −0.7) and AST was lower by −0.9 multiple of normal (95% CI: −1.1, −0.7).

An adjusted repeated measures analysis was performed in treated patients in the Fixed Complete Cohort to assess potential correlations between liver outcomes and age at baseline, genotype, the interaction of age at baseline and treatment timepoint, and the interaction of age at baseline and genotype. Age at baseline and the interaction between age at baseline and treatment timepoint were not correlated with ALT multiples of normal (Table 3). However, the interaction between age at baseline and genotype (using the most commonly occurring genotype, c.[894G>A];[894G>A] as the reference) was correlated with ALT multiples of normal in treated patients in the Fixed Complete Cohort (*p* = 0.0475; Table 3). There were no significant correlations between AST multiples of normal and any factor assessed (Table 3).

**TABLE 3:** Adjusted repeated measures analysis among treated patients in the Fixed Complete Cohort at baseline.

	ALT multiple of normal (*n* = 75)		AST multiple of normal (*n* = 70)	
	Estimate (95% CI)	*p*	Estimate (95% CI)	*p*
Treatment timepoint (baseline used as reference)		0.0015		< 0.0001
Result at 1 year	−1.4 (−2.0, −0.7)	0.0001	−0.8 (−1.3, −0.4)	0.0002
Result at 2 years	−1.0 (−1.5, −0.4)	0.0007	−0.8 (−1.2, −0.4)	0.0003
Result at 3 years	−0.9 (−1.5, −0.2)	0.0104	−0.9 (−1.2, −0.5)	< 0.0001
Age at baseline	0.0 (−0.1, 0.1)	0.9858	0.0 (−0.1, 0.0)	0.2926
Genotype (homozygous for c.894G>A used as reference)		0.0277		0.7638
Homozygous for other variants	2.3 (0.7, 3.8)	0.0047	0.5 (−0.7, 1.8)	0.4000
Compound heterozygous for c.894G>A	0.0 (−0.5, 0.6)	0.8593	−0.1 (−0.5, 0.4)	0.7910
Compound heterozygous for other variants	−0.3 (−1.3, 0.7)	0.5041	−0.2 (−1.0, 0.6)	0.6344
Age at baseline × treatment timepoint (baseline used as reference)		0.4291		0.7015
Result at 1 year	0.0 (0.0, 0.0)	0.7108	0.0 (0.0, 0.0)	0.9645
Result at 2 years	0.0 (−0.1, 0.0)	0.2726	0.0 (0.0, 0.0)	0.6873
Result at 3 years	0.0 (−0.1, 0.0)	0.3124	0.0 (0.0, 0.0)	0.9326
Age at baseline × genotype (homozygous for c.894G>A used as reference)		0.0475		0.5984
Homozygous for other variants	−0.2 (−0.3, 0)	0.0235	0.0 (−0.2, 0.1)	0.4966
Compound heterozygous for c.894G>A	0.0 (0.0, 0.1)	0.2600	0.0 (0.0, 0.0)	0.3109
Compound heterozygous for other variants	0.0 (−0.1, 0.2)	0.3999	0.0 (−0.1, 0.1)	0.8226

Abbreviations: ALT, alanine aminotransferase; AST, aspartate aminotransferase.

Of 22 untreated patients in the Fixed Complete Cohort with ALT results, 16 (73%) had ALT levels above the ULN at baseline (Figure S2A). Four of these 22 patients had ALT levels WNL after 1 year of follow‐up, and 6 (27%) had ALT levels WNL after 3 years (Figure S2A). Of 19 untreated patients in this cohort with AST results, 11 (58%) had AST levels above the ULN at baseline (Figure S2B). Seven of 19 (37%) patients had AST levels WNL after 1 year (Figure S2B); results were sustained over follow‐up. There was no appreciable change in ALT or AST multiples of normal over time in untreated patients from this cohort (Figure S3A,B).

### 3.4 ALT and AST After 5 Years of Follow‐Up

**FIGURE 4:**
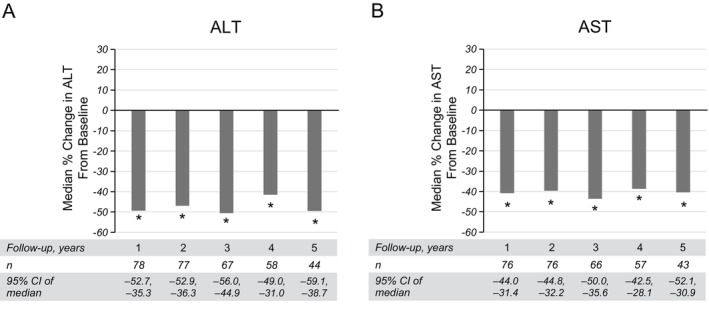
Percent change in ALT and AST over 5 years of follow‐up in patients treated with sebelipase alfa. *Significant based on 95% CIs. ALT, alanine aminotransferase; AST, aspartate aminotransferase.

While the Fixed Complete Cohort had a large number of patients assessed through 3 years of follow‐up, the number of patients with results through 5 consecutive years was considerably smaller. Thus, changes in aminotransferase levels over 5 years of follow‐up were analysed in a discrete cohort of patients. Cohorts of patients at each year of follow‐up were reported based on the availability of aminotransferase data at baseline and analysis year. Among treated patients with aminotransferase results at baseline and at 1 year of follow‐up, median percent changes in ALT and AST levels were −49% (95% CI: −52.7, −35.3; *n* = 78) and −41% (95% CI: −44.0, −31.4; *n* = 76), respectively (Figure 4A,B). Similar significant decreases were found in each cohort over 5 years of follow‐up. Among untreated patients with baseline data, changes in ALT and AST levels did not follow a consistent pattern over time (Figure S4A,B).

### 3.5 Safety

A total of 137 patients treated with sebelipase alfa were included in the safety analysis. The frequency of AEs in treated patients is summarised in Table 4. Throughout the study, 80 of 137 patients (58%) experienced at least 1 AE, which were mostly mild to moderate in severity, and 7 (5%) experienced infusion‐associated reactions. One infusion‐associated reaction (nonsevere anaphylactic reaction that resolved the same day) was deemed serious and treatment related. There were a total of 18 SAEs in 16 patients during follow‐up. All SAEs besides the nonsevere anaphylactic reaction were deemed non–treatment related and included pyrexia and appendicitis occurring in 2 patients each (1 of whom had acute ulcerated appendicitis) and infectious mononucleosis, laryngeal squamous cell carcinoma, renal colic, dyspnea, tracheitis, pneumonia, arthropathy, juvenile spondyloarthritis, syncope, tonsillitis, meningitis herpes, *Clostridium difficile* colitis, and hepatocellular carcinoma occurring in 1 patient each. One patient experienced febrile seizures on 3 separate occasions; however, none of these events occurred during registry follow‐up. No deaths occurred during follow‐up.

**TABLE 4:** Adverse events among patients treated with sebelipase alfa.

Adverse event description, *n* (%), number of events	Patients (*n* = 137)^a^
Patients with ≥ 1 AE	80 (58.4), 280
Patients with ≥ 1 infusion‐associated reactions	7 (5.1), 22
Patients with ≥ 1 SAE	16 (11.7), 18
Patients with ≥ 1 treatment‐related SAE	1 (0.7), 1
Deaths	0

Abbreviations: AE, adverse event; SAE, serious adverse event.

^a^ Safety population (*n* = 137) includes all patients enrolled in the registry who had ever been treated with sebelipase alfa.

## 4 Discussion

This analysis of the International LAL‐D Registry assessed ALT and AST levels over time as indicators of liver injury in patients with LAL‐D and the clinical impact of enzyme replacement therapy with sebelipase alfa on aminotransferase levels among treated patients. Treated patients were diagnosed at an earlier age than untreated patients and had more pronounced elevations of ALT and AST levels above the ULN at baseline. Treated patients had significant improvements in ALT and AST levels over time, with results sustained for up to 5 years of follow‐up. Untreated patients had fluctuations in ALT and AST levels over time. Overall, these findings suggest that treated patients have a more severe phenotype than untreated patients and that sebelipase alfa treatment normalises aminotransferase levels among patients who are at increased risk of progressing to liver disease in real‐world practice.

ALT and AST are both commonly measured in patients with LAL‐D [11, 21, 22, 26]. Evaluation of aminotransferases, particularly ALT, is also relevant for the assessment of patients with any of a wide array of other hepatic conditions, including metabolic dysfunction−associated steatotic liver disease, autoimmune hepatitis, and hepatitis B infection [27]. The US Food and Drug Administration recommends including ALT as an efficacy endpoint during the development of novel therapies for hepatitis B infection, highlighting the important role of this aminotransferase in monitoring liver outcomes [28]. Aminotransferases are also routinely assessed in comprehensive metabolic panels, making them an accessible outcome for monitoring disease progression.

The current analysis shows that paediatric patients diagnosed with LAL‐D have a more severe phenotype (as demonstrated by more pronounced elevations in ALT and AST levels) than patients diagnosed in adulthood. There was a significant inverse correlation between age and aminotransferase levels. The data presented in this manuscript are in line with the results of previous literature indicating that diagnosis of LAL‐D at a young age is associated with a more severe phenotype [1, 18]. A previous analysis of patients in the International LAL‐D Registry also showed an inverse relationship between patient age and fibrosis score, with patients < 12 years of age having more advanced fibrosis than those ≥ 12 years of age, collectively suggesting that patients with LAL‐D who present at an earlier age have more severe liver outcomes than adult patients [25].

The current analysis also shows that aminotransferase levels improved in paediatric and adult patients with LAL‐D who were treated with sebelipase alfa in real‐world practice. In all age categories, sebelipase alfa treatment increased the proportion of patients with aminotransferase levels WNL after 1 year of follow‐up. In the Fixed Complete Cohort, which included patients with baseline aminotransferase values above the ULN and data from 3 consecutive years of follow‐up, 41% had ALT and AST results WNL after 1 year. These results show that sebelipase alfa treatment results in normalisation of AST and ALT levels in more severely affected patients who were treated in real‐world practice. These findings are of particular interest since they provide a better understanding of changes in liver assessments over time in patients with regular monitoring, especially for those treated with sebelipase alfa. The findings concur with those of a previous randomised clinical trial and open‐label extension of sebelipase alfa in 59 children and adults with LAL‐D in which ALT levels normalised after treatment in 47% of patients [21]. Among treated patients in this study, ALT and AST levels were reduced from baseline by 49% and 41%, respectively, after 1 year of follow‐up, and reductions were similar in each unique cohort assessed over 5 years of follow‐up. Sebelipase alfa treatment decreased ALT and AST levels to a similar extent in open‐label extension studies with 3 to 5 years of follow‐up [5, 21, 22]. Although the number of patients analysed at each time point over the 5‐year follow‐up in this study varied because of real‐world data collection, the observed decreases in ALT and AST support the ongoing beneficial effect of sebelipase alfa treatment. Overall, the results of this manuscript confirm the results found in clinical trials and may provide reassurance to clinicians that sebelipase alfa treatment is effective in routine clinical practice.

This study has limitations. While it reports outcomes in treated and untreated patients, these 2 cohorts had markedly different clinical features at baseline; therefore, results in the two groups have limited comparisons with one another. As such, this study does not have a comparator arm and instead uses longitudinal measures of ALT and AST to characterise treatment effectiveness. The registry contains data only from real‐world follow‐up of patients. While data on liver or cardiac events (e.g., liver fibrosis/cirrhosis, portal hypertension, ischemic heart disease) are documented as part of real‐world patient follow‐up, these outcomes are infrequent, thus limiting extrapolation of ALT or AST data to clinical outcomes. While the registry collects data on LAL enzyme activity at diagnosis, enzyme activity is not routinely measured over time in clinical practice. As such, only baseline enzyme activity is available among registry patients. However, the registry does have complete and robust data on other variables, including demographics, genetic test results, and treatment status. The number of patients with data throughout follow‐up is variable because data are collected under routine clinical care in real‐world settings. Some analyses were performed on small sub‐populations of patients, potentially limiting interpretation of the results. Consistent with the known demographics of patients with LAL‐D, this study population primarily included White patients (93%).

## 5 Conclusions

The results of this registry analysis contribute evidence to a growing body of literature on the natural history of LAL‐D and the effectiveness of ERT with sebelipase alfa. While most patients had ALT and AST levels above the ULN at baseline, patients who received sebelipase alfa treatment were more likely to have pronounced ALT and AST elevations at baseline than those who were untreated. Treated patients with ALT and AST levels above the ULN at baseline had significant reductions in both measures over time, leading to normalisation of each aminotransferase level in 41% of patients after 1 year of treatment. These data suggest that enzyme replacement therapy with sebelipase alfa is likely to improve long‐term liver outcomes in patients with LAL‐D and warrant continued longitudinal investigation of LAL‐D in a larger cohort.

## Author Contributions

All authors contributed to the study and the development and/or subsequent revision of the manuscript. All authors read and approved the final manuscript for submission. The International LAL‐D Registry is sponsored by Alexion, AstraZeneca Rare Disease, and is overseen by a scientific advisory board that includes medical experts with experience in LAL‐D research or patient care.

## Ethics Statement

The International LAL‐D Registry (NCT01633489, EUPAS13276) protocol was approved by the institutional review board, or equivalent, at each participating site and was conducted in accordance with all applicable government regulations, the Declaration of Helsinki, and the principles of the International Council for Harmonisation Guidelines for Good Clinical Practice.

## Consent

All patients enrolled in the registry or their next of kin, if applicable, provided informed consent and authorization prior to submitting their data into the registry.

## Conflicts of Interest

Lorenzo D’Antiga, Emilio Ros, Manisha Balwani, Don P Wilson and William Balistreri are consultants/members of the Alexion, Astra Zeneca Rare Disease LAL‐d registry Advisory Board. Jennifer Evans and Florian Abel are employed by Alexion, Astra Zeneca Rare Disease.

## Supporting information


**Data S1:** liv70279‐sup‐0001‐Supinfo1.docx.

## Data Availability

Alexion, AstraZeneca Rare Disease will consider requests for disclosure of clinical study participant‐level data provided that participant privacy is assured through methods like data de‐identification, pseudonymization, or anonymization (as required by applicable law), and if such disclosure was included in the relevant study informed consent form or similar documentation. Qualified academic investigators may request participant‐level clinical data and supporting documents (statistical analysis plan and protocol) pertaining to Alexion‐sponsored studies. Further details regarding data availability and instructions for requesting information are available in the Alexion Clinical Trials Disclosure and Transparency Policy at https://www.alexionclinicaltrialtransparency.com/data‐requests/.
