# Supplementary material for: Sebelipase Alfa Improves Aminotransferase Levels in Lysosomal Acid Lipase Deficiency: Data From an International Registry
Source: Liver Int. 2025 Aug 8;45(9):e70279. doi: 10.1111/liv.70279 (PMC12334869; doi:10.1111/liv.70279)
Supplement: Supplementary file 1 — Data S1: liv70279‐sup‐0001‐Supinfo1.docx. [file LIV-45-0-s001.docx]

**Supplemental Material**

**Sebelipase alfa improves aminotransferase levels in lysosomal acid lipase deficiency: data from an international registry**

Lorenzo D’Antiga, et al.

**Supplemental Figure 1.** Patient flow diagram.


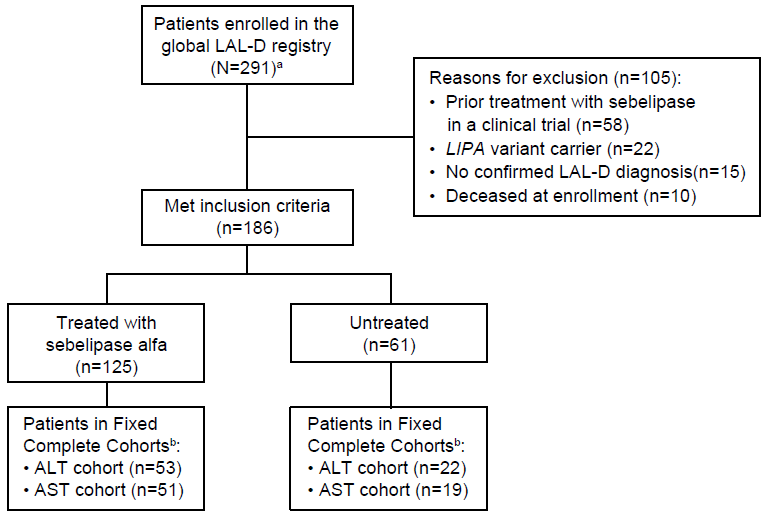


^a^Excludes 27 patients with rapidly progressive LAL-D who initiated sebelipase alfa treatment at <6 months of age. LAL-D, lysosomal acid lipase deficiency.

^b^Patients could be in both the ALT and AST cohorts.

**Supplemental Figure 2.** Proportion of untreated patients with ALT and AST values WNL at baseline and at 3 consecutive annual of follow-up measures.


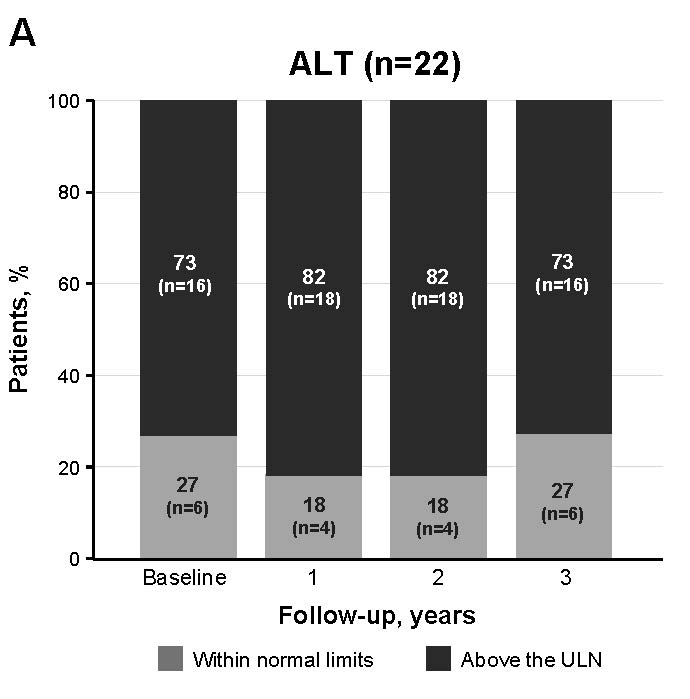

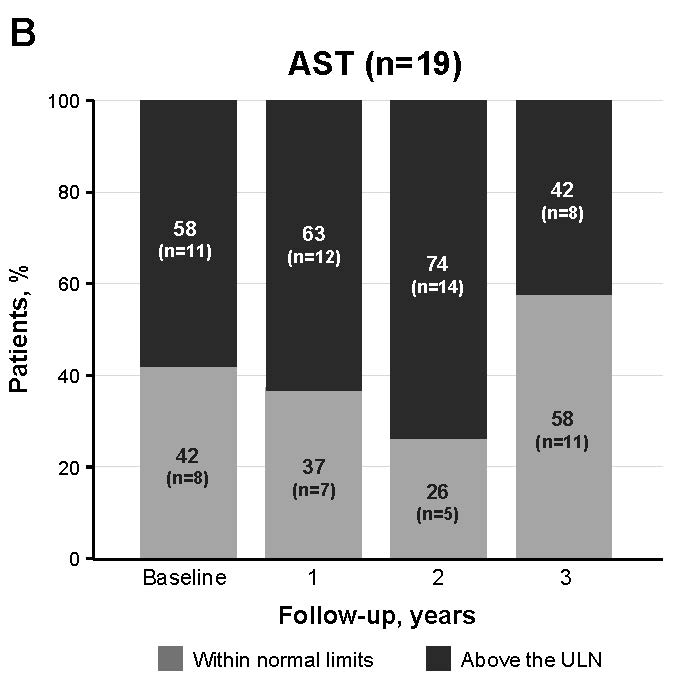


ALT, alanine aminotransferase; AST, aspartate aminotransferase; ULN, upper limit of normal; WNL, within normal limits.

**Supplemental Figure 3.** ALT and AST multiples of the ULN in untreated patients who had 3 consecutive annual follow-up measures.


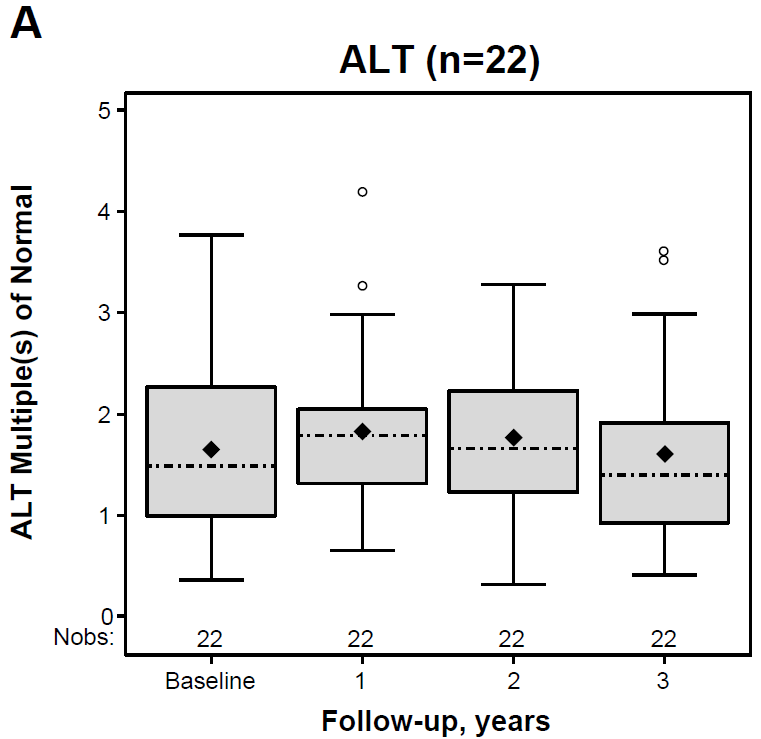

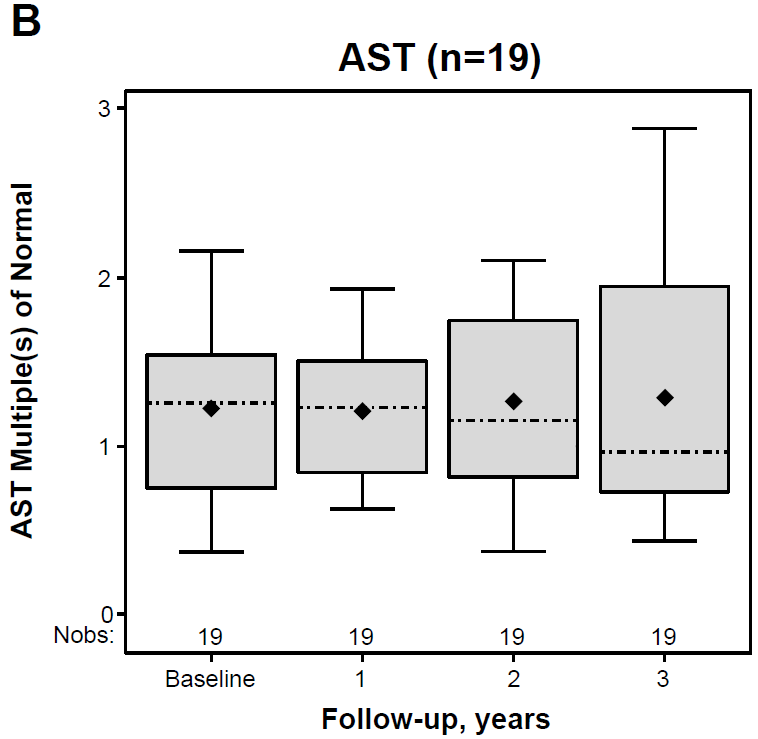


Open circles represent outliers, black diamonds represent means, dashed lines represent medians.

ALT, alanine aminotransferase; AST, aspartate aminotransferase; Nobs, number of observations; ULN, upper limit of normal.

**Supplemental Figure 4.** Percent Change in ALT and AST over 5 years of follow-up in untreated patients.


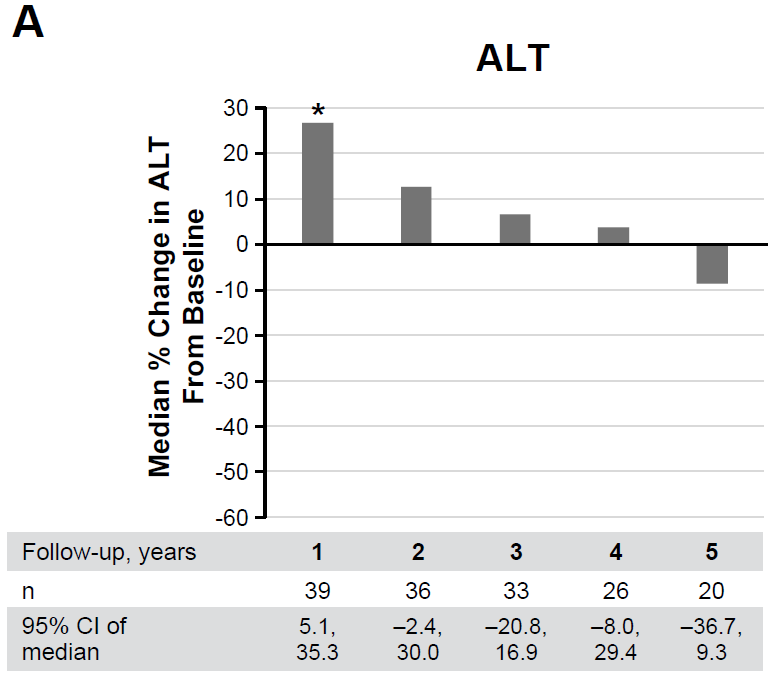

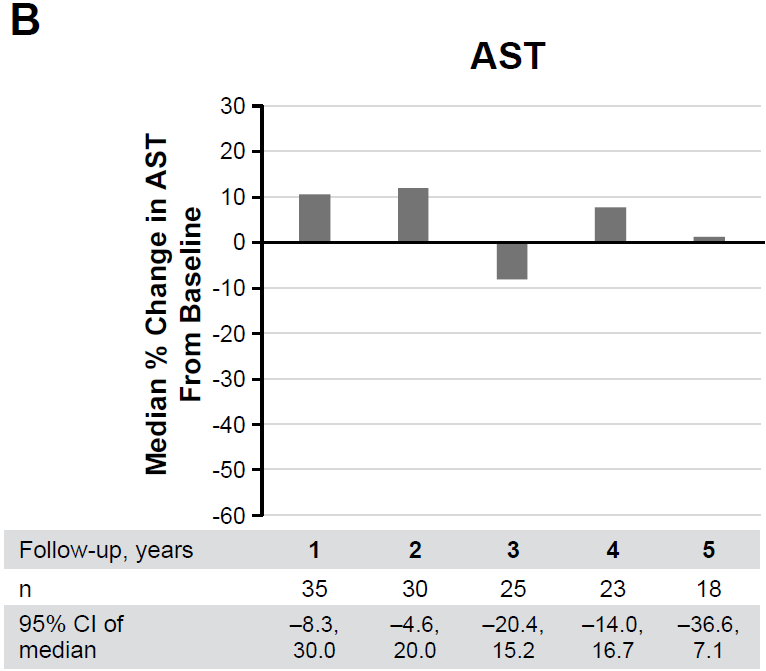


*Significant based on 95% CIs.

ALT, alanine aminotransferase; AST, aspartate aminotransferase; CI, confidence interval.

**Supplemental Table 1.** Baseline characteristics of patients treated with sebelipase alfa in the Fixed Complete Cohort.

| **Description** | **ALT Cohort**  **(n=53)** | **AST Cohort**  **(n=51)** |
| --- | --- | --- |
| Male, n (%) | 35 (66.0) | 34 (66.7) |
| Race, n (%) |  |  |
| White | 51 (96.2) | 49 (96.1) |
| Other/multiple | 2 (3.8) | 2 (3.9) |
| Age at diagnosis, median (Q1, Q3), years | 10.2 (6.4, 12.3) | 10.1 (6.3, 12.3) |

ALT, alanine aminotransferase; AST, aspartate aminotransferase; Q, quartile; ULN, upper limit of normal.
